# A goal-systems perspective on plant-based eating: keys to successful adherence in university students

**DOI:** 10.1017/S1368980020000695

**Published:** 2021-01

**Authors:** Maricarmen Vizcaino, Linda S Ruehlman, Paul Karoly, Katy Shilling, Andrew Berardy, Sidney Lines, Christopher M Wharton

**Affiliations:** 1 Nutrition Program, Radical Simplicity Lab, College of Health Solutions, Arizona State University, Phoenix, AZ 85004, USA; 2 Goalistics, LLC, Tempe, AZ 85287, USA; 3 Department of Psychology, Arizona State University, Tempe, AZ 85287, USA; 4 Swette Center for Sustainable Food Systems, Arizona State University, Tempe, AZ 85281, USA; 5 Department of English, University of British Columbia, Vancouver, BC Canada

**Keywords:** Plant-based diets, Vegetarianism, Veganism, Pescatarianism, Goals, Goals system assessment battery, Motivations

## Abstract

**Objective:**

To explore adherence to a plant-based diet from the perspective of goals- and motivations-based systems.

**Design:**

A cross-sectional, survey-based study was conducted regarding eating patterns, goals and motivations for current eating habits.

**Setting:**

Data were collected using an online survey platform, including the Goal Systems Assessment Battery (GSAB) and other survey tools.

**Participants:**

University students were recruited, including thirty-three students reporting successful maintenance of a plant-based diet (Adherents) and sixty-three students trying to adhere to a plant-based diet (Non-adherents).

**Results:**

Using GSAB subscale scores, discriminant function analyses significantly differentiated adherents *v*. non-adherents, accounting for 49·0 % of between-group variance (*χ*^2^ (13) = 42·03, *P* < 0·000). It correctly classified 72·7 % of adherents and 88·9 % of non-adherents. Constructs including value, self-efficacy, planning/stimulus control and positive affect were significant and included in the discriminant function. Logistic regression results suggested that participants who successfully adhered to a plant-based diet were seventeen times more likely to report ‘To manage or treat a medical condition’ as motivation and almost seven times more likely to report ‘To align with my ethical beliefs’ as motivation compared with non-adherents. However, these participants were 94 % less likely to report ‘To maintain and/or improve my health’ as motivation compared with non-adherents. Controlling for motivations, hierarchical logistic regression showed that only planning as part of the GSAB self-regulatory system predicted adherence to a plant-based diet.

**Conclusions:**

Values-based approaches to plant-based diets, including consideration for ethical beliefs, self-efficacy and proper planning, may be key for successful maintenance of this diet long-term.

Interest in plant-based diets continues to grow with increasing market availability of alternative protein products as well as consumer demands for foods that are perceived to be healthful, ethical and sustainable^(1,2)^. Common plant-based dietary patterns include veganism (total elimination of animal-based foods), lacto-ovo-vegetarianism (which excludes animal-based foods other than eggs and dairy), flexitarianism (which is generally vegetarian but rarely to occasionally includes meat or fish) and pescatarianism (which excludes animal-based foods other than eggs, dairy and fish)^(3,4)^. The health benefits provided by plant-based diets are abundant, notably including more consistent weight management, reduced blood pressure and improved lipid profile^(5–8)^. These and other improved markers of health likely contribute to the role plant-based diets may play in decreasing risk for all-cause mortality, lower prevalence of metabolic syndrome and diabetes, lower incidence of heart disease and total cancers and decreased odds for developing overweight and obesity over time^(9–11)^. And while plant-based diets can be effective in preventing multiple chronic diseases, they may also contribute to improved health status of those already diagnosed with a chronic condition such as type 2 diabetes or osteoarthritis^(12–15)^.

Health benefits of plant-based dietary patterns are not reserved exclusively to strict vegan/vegetarian diets. They can also be obtained from diets that not only remain plant heavy but also include modest intakes of fish, dairy consumption and occasional meat (e.g. pescatarian, flexitarian and Mediterranean diets)^(16)^. For instance, significantly lower odds of overweight/obesity, high TAG and high LDL were recently noted among semi-vegetarians compared with non-vegetarians in a South Asian sample^(11)^.

The benefits of plant-based diets extend beyond issues of human health as well; they have featured prominently, for example, in discussions regarding environmental sustainability. Plant-based diets have the potential to reduce impacts on the environment and contribute to improved food system resilience over time in multiple ways. A large body of research shows that meat-heavy diets require greater amounts of natural resources compared with plant-based diets. For example, on average, about 104·6 kJ (25 kcal) of fossil fuel energy are required to produce 4·184 kJ (1 kcal) of animal protein, compared with 9·2048 kJ (2·2 kcal) of fossil fuel energy to produce 4·184 kJ (1 kcal) of plant protein^(17)^. Beef production specifically is even more intensive, requiring forty times more energy^(18)^. One recent study also demonstrated that beef was the least efficient among commonly consumed protein foods when considering both global warming potential and protein quality simultaneously^(19)^. And Cordell *et al.* ^(20)^ showed that meat-based diets required nearly three times the phosphorus as plant-based diets.

In addition to health and environmental motives, ethical concerns also drive the choice of plant-based diets^(21,22)^. One recent study demonstrated that empathy towards humans and animals was associated with positive attitudes towards plant-based dishes, in particular among vegetarians and flexitarians^(23)^. Other research has shown that vegetarians who were motivated by concerns for animals identified their dietary choices as a mechanism by which they could achieve more socially and morally oriented goals^(24)^.

Despite compelling benefits and a host of motivations to pursue plant-based diets, only 2–5 % of American adults are following a vegetarian diet and only 0·5–2 % consume no animal products at all^(25–27)^. Furthermore, the prevalence of vegetarians and vegans in the USA has not changed in the last 15 years^(25)^ despite the continuously growing market for plant-based products^(1,2)^. In fact, Americans generally fail to consume even the minimum recommendations for fruit and vegetable consumption, with only one in ten adults meeting recommendations^(28)^ based on the 2015–2020 Dietary Guidelines for Americans^(29)^.

Adopting and then sustaining a vegetarian or vegan diet can be challenging. The Humane Research Council found five times more former vegetarians/vegans than current vegetarians/vegans in the USA^(30)^. Moreover, former vegetarians/vegans do not sustain their diet for very long; approximately 34 % followed their diet for 3 months, whereas 53 % adhered to their diet for <1 year^(30)^. In addition, even those who were identified as vegetarian do not consistently adhere to their dietary goals; in a previous study using a representative sample of the USA, only a fifth of self-identified vegetarians were consistent in pursuing a strictly vegetarian diet^(31)^.

It is hard to overstate the difficulties for the adoption and adherence of vegetarian and vegan diets specifically. Change occurs – or fails to occur – in the context of a complex motivational system reflecting personal, social and environmental constraints and affordances^(32–35)^. Previous studies investigating the characteristics of individuals who decide to follow a vegetarian/vegan diet have revealed a host of motivational factors including a desire to enhance personal well-being and/or health, treatment of a specific health problem, ethical concerns (e.g. animal welfare and environment protection), taste preferences, religious beliefs and social/family motives^(27,30,36–39)^. Nonetheless, much less is known about the control mechanisms that allow individuals to sustain a vegetarian/vegan diet in the face of numerous obstacles.

The desire to initiate and sustain a plant-based diet can be seen as a goal motivated by any of the above reasons. Goals are defined as internal representations of desired states that compel the individual to decrease the discrepancy between a desired state and the current state^(40)^. After a person has determined the goals to be pursued and their respective success criteria, the process of goal striving begins^(41)^. Successful goal striving will depend on overcoming two primary self-regulatory challenges: the planning and execution of actions that promote goal achievement and the protection of valued goals from disruption given the likely presence of competing goals^(41)^.

The present study included examination of how people think about, appraise or cognitively frame the process of goal striving towards a plant-based diet in an attempt to capture the ‘internal workings’ or the ‘how’ of the motivational system. A major focus was on ‘goal representation’ as elaborated in Ford’s *living systems model* of human self-directedness^(42)^. Ford’s model postulates a goal-based self-regulating system that comprises a set of basic organising functions. The *directive* or *feedforward function* taps the thoughts or beliefs that presumably activate the individual to move towards a particular goal or end state – this function establishes *what* the individual desires. The *regulatory function* serves as a ‘comparator’ mechanism, evaluating how well the current state matches the desired state. The *control function* institutes strategies to correct discrepancies between the current and desired state. Finally, the *arousal function* provides the emotional activation for goal-directed behaviour.

Thus, following Ford’s model, the planning, regulation and execution of behaviour that support the goal of adhering to a plant-based diet were examined. In addition, because ethical/moral motivations have been shown to be common among those following a vegan/vegetarian diet^(21–24,36–39)^, we examined the role of type of motivations on the likelihood of adhering to a plant-based diet, alone and in conjunction with the elements of the self-regulatory system outlined above.

In sum, the study investigated the process of successful striving towards the adherence of a plant-based diet using a goal-systems perspective that captures the internal workings of the motivational system. Hypotheses were as follows:

H1: Individuals who successfully adhere to a plant-based diet (Adherents) will show a more effective self-regulatory system compared with individuals who struggle to adhere to a plant-based diet (Non-adherents). Specifically, it was expected that adherents would display (1) significantly higher levels of value, self-efficacy, self-monitoring, social comparison, planning, self-reward and positive affect and (2) significantly lower levels of self-criticism and negative affect, compared with non-adherents.

H2: Type of motivations will significantly predict adherence to a plant-based diet.

H3: Elements of the self-regulatory system, such as value, self-efficacy and planning, will predict adherence to a plant-based diet controlling for motivations.

## Methods

### Participants

Participants in the present study were recruited from a large online survey conducted with students enrolled in an Introduction to Psychology course in a university of the Southwest of the United States. The study was approved by the university’s Institutional Review Board. Students received research credits for their Introduction to Psychology course in compensation for their participation in the study. This original sample comprised 1501 students with a mean age of 19·24 years (sd 2·55). Fifty-four percentage were female; 54 % were White, 4 % African American, 18 % Hispanic/Latino, 1 % Native American, 10 % East Asian, 3 % South Asian, 4 % Asian American and 6 % other background.

In the initial screening survey, students were asked about their current eating preferences with a multiple-choice question. The options were: (1) vegan for 12 months or longer, (2) vegetarian for 12 months or longer, (3) pescatarian for 12 months or longer, (4) currently striving to change eating habits in order to achieve a vegan, vegetarian or pescatarian diet, but not always successful, (5) past unsuccessful attempts to achieve a pescatarian, vegetarian or vegan diet and currently having no desire to try again and (6) never tried to achieve a vegan, vegetarian or pescatarian diet with no desire to do so. A standard definition for each type of diet was provided (e.g. ‘I would describe myself as vegan. I have not eaten meat, seafood, poultry, dairy, or eggs for 12 months or longer.’).

Students who described themselves as vegan, vegetarian or pescatarian for 12 months or longer were designated to the Adherents group (*n* 91; 6·1 %), whereas those who described themselves as currently trying to adhere to a plant-based diet but were not always successful were designated to the Non-adherents group (*n* 178; 11·9 %). Students from these two groups were recruited via email to participate in a follow-up survey. A total of thirty-three students from the Adherent group and sixty-three from the Non-adherent group provided informed consent and completed the follow-up survey. No statistical differences in demographic characteristics were found between those students who agreed to participate in the follow-up survey and those who did not agree (all *P* > 0·11).

### Follow-up survey

The follow-up survey was implemented through Qualtrics and included questions about demographic background and the variables under investigation. Completion of the survey took approximately 8–10 min. Demographic questions included age, sex, race/ethnic and religious affiliation, political views and socio-economic status (please see Table 1).

**Table 1:** Demographic characteristics of non-adherents and adherents to a plant-based diet (*n* 96)

	Non-adherents (*n* 63)	Adherents (*n* 33)	*P* value
Age (years)			
Median	18	18	0·993
Interquartile range	18–19	18–19·5	
Sex (%)			
Male	30·6	30·3	0·972
Ethnicity (%)			
Caucasian	46·0	51·5	0·701
African-American	7·9	3·0	
Hispanic	17·5	12·1	
Native American	1·6	0·0	
Other	27·0	33·3	
Religious affiliation (%)			
Atheist	11·5	9·1	0·539
Agnostic	11·5	15·2	
Catholic	14·8	9·1	
Christian, but not Catholic	27·9	18·2	
Spiritual, but not religious	21·3	21·2	
Other	13·1	27·3	
Political views (%)			
Democrat	58·1	51·5	0·808
Republican	14·5	15·2	
Other	27·4	33·3	
Socio-economic status (%)			
Upper class	6·3	9·1	0·499
Upper-middle class	30·2	42·4	
Middle class	42·9	30·3	
Lower-middle class	14·3	18·2	
Working class	4·8	0·0	
Other	1·6	0·0	

* Age is presented as medians and interquartile ranges due to non-normality. *P* value represents results from *χ* ^2^ analyses for categorical data and Mann–Whitney *U* test for continuous data.

Goal representation during the process of goal striving towards a plant-based diet was assessed with the Goal Systems Assessment Battery (GSAB)^(43)^, a thirty-six-item self-report questionnaire based upon Ford’s living systems model of human self-directedness^(42)^ that taps the four previously described functions of a self-regulating system. These functions are measured via nine subscales that include value and self-efficacy – *directive or feedforward function*; social comparison and self-monitoring – *regulatory function*; planning/stimulus control, self-reward and self-criticism – *control function*; and positive affect and negative affect – *arousal function*.

An example of an item for the self-monitoring subscale would be: ‘I keep track of my overall progress toward this goal’, whereas an example of an item for the social comparison subscale would be ‘I evaluate my progress toward this goal in comparison to how well other people are doing in pursuing it’. Each item is answered in a Likert scale ranging from 0 = not at all to 4 = extremely. Subscale scores are derived from the sum of the items associated with each particular subscale; scores can range from 0 to 16. Higher scores represent a higher degree of the concept under study. Participants in the present study were instructed to respond to each GSAB item in relation to their goal of achieving a plant-based diet (either vegan, vegetarian or pescatarian).

The GSAB has been used in a variety of research settings and populations, possesses acceptable psychometric properties including good retest reliability, low social desirability response bias and ample evidence of predictive validity^(43)^. In addition, the factor structure of the instrument has been previously confirmed (comparative fit index = 0·91–0·99)^(43)^. Research has shown that the GSAB relates in a statistically significant manner to mental health functioning, exercise participation, BMI and pain experience^(43–48)^.

Lastly, students were asked to indicate their motivations for their current dietary pattern from a list of thirteen possible motivations. Students were free to select multiple motivations and were also provided an option to enter ‘other’ motivation not included in the list (please see Table 4). This list of motivations was created based on findings from previous research^(21–24,36–39)^.

### Statistical analyses

Statistical analyses were performed with the Statistical Package for the Social Sciences version 24 (IBM). Preliminary *χ* ^2^ and Mann–Whitney *U* test analyses were conducted for categorical and continuous variables, respectively. No significant demographic differences between groups were found (see results section below), and hence demographics were not included in subsequent analyses.

To test our primary hypothesis (H1) that Adherents would show a more effective self-regulatory system compared with Non-adherents, a discriminant function analysis using the nine GSAB subscales was conducted to examine if groups can be differentiated by a unique combination of self-regulatory scores. Initial data screening indicated no missing data and no violations of statistical assumptions, except for a few univariate outliers; these outliers, which were determined to potentially bias populations parameters, were removed as suggested by the literature^(49)^, resulting in a total sample of eighty-nine participants whose data were used for this analysis.

To test our second hypothesis (H2), we conducted a logistic regression analysis to assess whether a variety of motivations for a plant-based diet predicted the likelihood of successfully adhering to a plant-based diet goal.

Finally, to test our third hypothesis (H3), we conducted a hierarchical logistic regression to assess whether elements of the self-regulatory system predict the likelihood of adhering to a plant-based diet when controlling for motivations. Significance was set at *α* < 0·05.

## Results

*χ* ^2^ analyses revealed no significant differences between groups for sex, *χ* ^2^ (1, *n* 95) = 0·001 and *P* = 0·97; religion, *χ* ^2^ (5, *n* 94) = 4·07 and *P* = 0·53; race/ethnic affiliation, *χ* ^2^ (4, *n* 96) = 2·18 and *P* = 0·70; political views, *χ* ^2^ (2, *n* 95) = 0·42 and *P* = 0·80; or socio-economic status, *χ* ^2^ (5, *n* 96) = 4·36 and *P* = 0·49. Similarly, the Mann–Whitney *U* test indicated no difference in age between groups; *U* = 1038·5, *z* = –0·009, and *P* = 0·99.

The discriminant function calculated based on the combination of the GSAB scores significantly differentiated adherents *v*. non-adherents and accounted for 34·7 % of the between-group variance, *χ* ^2^ (9) = 35·20, *P* < 0·000 (please see Table 2 for GSAB scores). It correctly classified 71·4 % of the adherents and 80·3 % of the non-adherents. Value, self-efficacy, planning/stimulus control and positive affect were positively correlated with the discriminant function, whereas self-monitoring and self-criticism were negatively correlated. Nonetheless, only value, self-efficacy, planning/stimulus control and positive affect showed correlations higher than 0·33, which is considered acceptable for inclusion in the discriminant function^(49)^. Social comparison, self-reward and negative affect did not significantly contribute to the distinction between groups. Please refer to Table 3.

**Table 2:** Participants’ scores on the different subscales of the Goal Systems Assessment Battery (GSAB) instrument

Subscale	Non-adherents			Adherents		
	Median		Interquartile range	Median		Interquartile range
Value	12		8–14	14·5		11–16
Self-efficacy	11		9–13	14		11–15
Social comparison	6		2–9	6		1·5–9
Self-monitoring						
Mean		8·93			10·18	
sd		3·11			2·55	
Planning						
Mean		8·25			10·96	
sd		3·25			2·60	
Self-criticism	7		3–10	5		2–8
Self-reward						
Mean		8·17			7·27	
sd		3·90			4·32	
Positive affect	10		6–12	12		8·5–13·5
Negative affect	4		1–7	4		2·5–7·5

* Means and standard deviations are presented for normally distributed data. Medians and interquartile ranges are presented for non-normally distributed data. Total range of scores for all subscales above was 0–16.

**Table 3:** Standardized discriminant function coefficients and structure matrix correlations in the discriminant function analysis using the Goal Systems Assessment Battery (GSAB) subscales to differentiate between adherents and non-adherents to a plant-based diet

Subscale	Coefficient	*R*	*r* ^2^ (%)	*P* value
Value	0·173	0·414	17·13	0·**006**
Self-efficacy	0·112	0·361	13·03	0·**016**
Social comparison	–0·002	0·312	09·73	0·989
Self-monitoring	–0·164	–0·002	00·00	0·**037**
Planning	0·786	0·602	36·24	0·**000**
Self-criticism	–1·028	–0·306	09·36	0·**040**
Self-reward	–0·366	–0·071	00·50	0·630
Positive affect	0·039	0·375	14·06	0·**012**
Negative affect	0·913	0·122	01·48	0·410

Significant *P* values are bolded.

The logistic regression analysis assessing motivations as predictors on the likelihood of adhering to a plant-based diet indicated that the model was significant, *χ* ^2^ (13) = 42·03, *P* < 0·000. The Hosmer and Lemeshow test indicated that the model described the data well (*χ* ^2^ (8) = 5·92, *P* = 0·66) correctly classifying 72·7 % of adherents and 88·9 % of non-adherents, whereas the Nagelkerke *R* ^2^ indicated that the model explained 49·0 % of the variance.

The only significant individual predictors were ‘To maintain and/or improve my health,’ ‘To manage or treat a medical condition’ and ‘To align with my ethical beliefs’. Participants who successfully adhered to a plant-based diet were seventeen times more likely to report ‘To manage or treat a medical condition’ as motivation and almost seven times more likely to report ‘To align with my ethical beliefs’ as motivation compared with non-adherents. On the contrary, participants who successfully adhered to a plant-based diet were 94 % less likely to report ‘To maintain and/or improve my health’ as motivation compared with non-adherents. (Table 4)

Only significant variables from the previous two analyses were included in the final hierarchical logistic regression analysis. Results indicated that model 2 assessing the significance of the self-regulatory system on predicting adherence to a plant-based diet controlling for motivations was significant, *χ* ^2^ (7) = 45·09, *P* < 0·000. The Hosmer and Lemeshow test indicated that model 2 adequately described the data (*χ* ^2^ (8) = 14·63, *P* = 0·07) correctly classifying 73·3 % of adherents and 88·9 % of non-adherents. The Nagelkerke *R* ^2^ indicated that explained variance increased from 39·5 % in model 1 (motivations only) to 53·7 % in model 2 (motivations + elements from self-regulatory system). However, the only significant predictor from the self-regulatory system was planning; those that scored higher on planning had a 37 % higher likelihood to adhere to a plant-based diet. Please refer to Table 5.

**Table 4:** Results of logistic regression analyses using motivations for current eating habits to predict the likelihood of successfully adhering to a plant-based diet

	Exp (B)	95 % CI	*P* value
To maintain and/or improve my health	**0·05**	**0·00**, **0·41**	0·**004**
To manage or treat a medical condition	**17·41**	**2·58**, **117·16**	0·**003**
To align with my ethical beliefs	**6·88**	**1·82**, **25·95**	0·**004**
To adhere to my religious beliefs	3·50	0·27, 45·12	0·336
To have the best tasting food	0·30	0·04, 1·99	0·216
To maximise my pleasure or enjoyment while eating	2·18	0·38, 12·35	0·375
To fit within my budget	0·60	0·14, 2·60	0·501
To support my local farmers and/or community	1·64	0·36, 7·32	0·514
To support sustainable practices	1·94	0·48, 7·78	0·349
To act like the person I want to be	2·55	0·73, 8·89	0·139
To conform with societal expectations	0·56	0·04, 7·20	0·663
To be true to myself and live in accordance with who I am	1·46	0·30, 7·13	0·638

Significant *P* values are bolded.

**Table 5:** Results of hierarchical logistic regression analysis predicting adherence to a plant-based diet from motivations and elements from the self-regulatory system

Variable	Model 1			Model 2		
	B	Exp(B)	95 % CI	B	Exp(B)	95 % CI
Constant	0·15	1·16		–3·72	0·024*	
To maintain and/or improve my health	–2·43	0·088**	0·01, 0·41	–2·97	0·051**	0·00, 0·31
To manage or treat a medical condition	2·39	10·97**	2·28, 52·65	2·38	10·81*	1·76, 66·15
To align with my ethical beliefs	2·35	10·49**	3·17, 34·66	2·27	9·76**	2·44, 39·02
Value				0·01	1·01	0·76, 1·35
Self-efficacy				0·12	1·13	0·84, 1·52
Planning				0·31	1·37*	1·06, 1·78
Positive affect				–0·04	0·95	0·76, 1·19

*P < 0·05, **P < 0·01.

## Discussion

The main purpose of this study was to examine factors related to successful striving towards the adherence of a plant-based diet using a goal-systems perspective. We hypothesised that individuals who successfully adhered to a plant-based diet would show a more effective self-regulatory system compared with individuals who struggled to adhere to a plant-based diet. The results partially supported our hypothesis. High levels of value, self-efficacy, planning/stimulus control and positive affect significantly contributed to the distinction between adherents and non-adherents; among these predictors, planning showed the highest correlation and explained more than 36 % of the difference between groups. These results reflect prior research related to health behaviours in college students, in which goal regulatory thinking was also strongly supportive of exercise goals among regular exercisers compared with irregular exercisers, for whom other life goals competed with attempts to exercise^(44)^. Other applications of goal regulatory thinking have been studied in this population in relation to academic performance, in which certain types of academic goals were associated with better or worse exam scores, mediated by self-regulatory skills^(50)^.

Thus, in the process of goal representation, the control function as represented by planning/stimulus control appears to have the greatest relevance among young adults striving to maintain a plant-based diet. Accordingly, careful and strategic planning may allow for a successful adjustment of inconsistencies between current and desired states. Social psychological theory postulates that the planning of actions promoting goal achievement is one of the primary self-regulatory challenges to overcome for successful goal striving^(41)^. In addition, planning is considered the key element that connects behavioural intentions and actions^(44)^. The results from this study support these concepts and highlight the important role of planning specifically for successful adherence to a plant-based diet.

This study also investigated the significance of motivations for predicting the likelihood of successfully adhering to a plant-based diet goal. We found that health and ethical motivations were significant predictors for adherence, which is in line with the current literature. Health benefits and ethical concerns are the most commonly cited reasons for starting a vegan diet^(37)^. However, we found that ‘To maintain and/or improve my health’ as a motivation was in fact predictive of a lesser likelihood of adhering to a plant-based diet. Similarly, the Humane Research Council found that former vegetarians often reported health concerns as the only reason for starting a plant-based diet^(30)^. On the contrary, the motivation to treat a medical condition significantly predicted the likelihood of successfully adhering to this type of dietary goal. Our study thus adds to the literature by making the distinction between following a plant-based diet for improving health in general *v*. caring for a specific medical condition, the latter appearing to have a stronger impact on successful goal striving.

While the medical community and general public may acknowledge the advantages of following a plant-based diet for treating a variety of medical conditions^(15)^, choosing to completely or partially eliminate animal products from one’s diet because of ethical reasons is worthy of further discussion. In a previous study, individuals who originally became vegetarian for ethical reasons had been vegetarian for significantly longer and showed a higher conviction compared with those who became vegetarian for health reasons^(51)^. Conversely, lower justice concerns (animal protection, environmental concerns and ending world hunger) have been found to fully mediate the relationship between conservatism and lapsing to meat-eating among individuals initially adopting a vegan/vegetarian diet^(52)^.

It has been suggested that following a plant-based diet for ethical reasons involves values for which food is another form of expression, and therefore, a plant-based diet is not the ultimate goal in itself but the means to attain a larger overall goal that derives from these values and a strong personal identity^(39)^. Thus, for followers of a plant-based diet, food is not just a source of nutrition but also perhaps reflective of who a person is and who that person would like to be^(53)^. Following a plant-based diet can then be seen as an element of an overall lifestyle framework that provides individuals with purpose and personal satisfaction. Related research offers some insights along these lines. Studies exploring the relation between Goldberg’s Big Five personality traits and dietary intake, for example, suggest that aspects that help to define one’s personality may influence food choices. One recent review noted the trait of conscientiousness as being particularly relevant in relation to health behaviours, specifically healthy eating^(54)^. Another large study in obese individuals identified conscientiousness, among other personality traits, as being related to restrained eating, which could be important in weight regulation over time^(55)^.

The results from our hierarchical logistic regression analysis also showed an increase from 39·5 to 53·7 % of explained variance when planning was added to motivations as predictors of adherence to a plant-based diet. Thus, the results from this study indicate that an individual must develop a coherent self-regulatory system that facilitates the process of striving towards a plant-based diet goal, in which planning appears to be essential. Planning is divided into two distinct constructs: action planning (the process of specifying the when, where and how to act) and coping planning (the mental representation of potential risk situations and appropriate coping responses). Action planning has been found more impactful in the early implementation of behaviour change, whereas coping planning has been found more impactful during behaviour maintenance^(56)^. Prior research has demonstrated that among the barriers for adopting a plant-based diet, it is the perception that it is difficult and time-consuming to prepare vegetarian-style foods, not knowing what to eat instead of meat, and not wanting to be stereotyped negatively^(57)^. Hence, in the context of striving towards a plant-based diet, action planning may involve learning how to cook vegetarian-style foods, preparing a shopping list and developing a weekly meal plan, whereas coping planning may entail mentally preparing for adverse social situations by appealing to personal values and convictions. Future research should focus not only on motivations for adopting a plant-based diet but also on strategies for effective planning in order to achieve a sustained dietary behaviour change.

### Limitations

Some of the limitations of this study include a relatively small sample size and the self-report nature of eating preferences. Future studies should corroborate adherence to a plant-based diet with detailed dietary logs and/or biological markers with larger samples. Future studies should also include participants that follow other forms of plant-based diets such as flexitarians/semi-vegetarians. In addition, our list of motivations to follow a plant-based diet, though reflective of the literature, is by no means exhaustive; other motivations may be significant and worthy of future investigation. Similarly, other psychological factors could be important in relation to interest in, and adhering to, plant-based diets that were not evaluated in this study. For example, conscientiousness as defined in Goldberg’s ‘Big Five’ personality traits could further explain the ways in which individuals’ motivations and sense of identity relate to food choices and dietary patterns. Lastly, the results of this study should be corroborated with different populations such as full-time working adults and individuals with varied demographic characteristics; the process of self-regulation and/or motivations may differ as work/family responsibilities change, whereas motivations, and in particular ethical beliefs, may be stronger among certain ethnic/religious groups.

## Implications for research and practice

Ethical beliefs and planning seem to be key elements for the successful implementation and maintenance of a plant-based diet. Plant-based diets represent important opportunities for improved health and environmental impact simultaneously. As such, future research as well as public health campaigns designed to promote more plant-heavy diets could potentially be more impactful if they leverage multiple motivators including ethical beliefs and sustainability concerns. Further, public health campaigns could improve success by incorporating guidelines and other tools focused on effective planning of both the implementation of plant-based diets as well as their maintenance over time and in multiple social situations.
